# 3D bioprinting of fish skin-based gelatin methacryloyl (GelMA) bio-ink for use as a potential skin substitute

**DOI:** 10.1038/s41598-024-73774-1

**Published:** 2024-10-05

**Authors:** Nuttapol Tanadchangsaeng, Kitipong Pasanaphong, Tulyapruek Tawonsawatruk, Kasem Rattanapinyopituk, Borwornporn Tangketsarawan, Visut Rawiwet, Alita Kongchanagul, Narongrit Srikaew, Thanaporn Yoyruerop, Nattapon Panupinthu, Ratirat Sangpayap, Anuchan Panaksri, Sani Boonyagul, Ruedee Hemstapat

**Affiliations:** 1 College of Biomedical Engineering, Rangsit University, Pathum Thani, Thailand; 2 Department of Orthopaedics, Faculty of Medicine Ramathibodi Hospital, Mahidol University, Bangkok, Thailand; 3 Department of Pathology, Faculty of Veterinary Science, Chulalongkorn University, Bangkok, Thailand; 4 Department of Pharmacology, Faculty of Science, Mahidol University, Bangkok, Thailand; 5 Central Animal Facility, Faculty of Science, Mahidol University, Bangkok, Thailand; 6 Center for Vaccine Development, Institute of Molecular Biosciences, Mahidol University, Bangkok, Thailand; 7 Research Center, Faculty of Medicine Ramathibodi Hospital, Mahidol University, Bangkok, Thailand; 8 Mahidol University-Frontier Research Facility (MU-FRF), Mahidol University, Nakhon Pathom, Thailand; 9 Department of Physiology, Faculty of Science, Mahidol University, Bangkok, Thailand; 10 Center of Calcium and Bone Research (COCAB), Faculty of Science, Mahidol University, Bangkok, Thailand

**Keywords:** Bio-ink, 3D bioprinting, Critical-sized full thickness skin defect, Fish skin gelatin, GelMA hydrogel, Skin substitute, Health care, Materials science, Mesenchymal stem cells, Regeneration

## Abstract

Gelatin methacryloyl (GelMA), typically derived from mammalian sources, has recently emerged as an ideal bio-ink for three-dimensional (3D) bioprinting. Herein, we developed a fish skin-based GelMA bio-ink for the fabrication of a 3D GelMA skin substitute with a 3D bioprinter. Several concentrations of methacrylic acid anhydride were used to fabricate GelMA, in which their physical-mechanical properties were assessed. This fish skin-based GelMA bio-ink was loaded with human adipose tissue-derived mesenchymal stromal cells (ASCs) and human platelet lysate (HPL) and then printed to obtain 3D ASCs + HPL-loaded GelMA scaffolds. Cell viability test and a preliminary investigation of its effectiveness in promoting wound closure were evaluated in a critical-sized full thickness skin defect in a rat model. The cell viability results showed that the number of ASCs increased significantly within the 3D GelMA hydrogel scaffold, indicating its biocompatibility property. In vivo results demonstrated that ASCs + HPL-loaded GelMA scaffolds could delay wound contraction, markedly enhanced collagen deposition, and promoted the formation of new blood vessels, especially at the wound edge, compared to the untreated group. Therefore, this newly fish skin-based GelMA bio-ink developed in this study has the potential to be utilized for the printing of 3D GelMA skin substitutes.

**Supplementary Information:**

The online version contains supplementary material available at 10.1038/s41598-024-73774-1.

## Introduction

Autologous skin grafts are considered the gold standard for treating large, full-thickness skin wounds caused by acute burns and other traumatic injuries; however, this practice possesses several limitations, including limited availability of donor skin grafts and a significant discomfort at the donor site^1^. This has driven the development of skin substitutes. With advancements in tissue engineering, several biomaterials have been explored for their potential use in fabrication. Hydrogels were the first biomaterials developed and used in clinical practice and remain commonly used among other biomaterials^2^. Hydrogels can be composed of natural polymers like gelatin, collagen, chitosan, and hyaluronic acid, or synthetic polymers such as poly (lactic acid) (PLA), poly (ethylene glycol), and poly (lactic-co-glycolic acid). These polymers are crosslinked through various physical or chemical methods for biomedical purposes such as regenerative medicine, tissue engineering, and drug delivery^3,4^. Hydrogels from naturally occurring biopolymers offer several benefits over synthetic polymers, including biocompatibility, minimal immunogenicity, and the ability to degrade quickly^5^. In addition, gelatin-based hydrogels loaded with adipose-derived mesenchymal stem cells (ASCs) have been shown to promote skin wound regeneration as they serve as a cell-seeded scaffold that maintains a favorable microenvironment for cell survival and proliferation^6^.

Recently, gelatin methacrylate (GelMA)-based hydrogel has become a more attractive material in the fields of tissue engineering and regenerative medicine^7,8^. GelMA-based hydrogels offer several advantages, including biocompatibility, biodegradability, low immunogenicity, and tunable physical and chemical characteristics. These properties can be customized to meet the requirements of various applications through adjustments in methacrylation degree and gel concentration^9,10^. In addition, GelMA hydrogel preserves natural, specific cell-binding sites that are essential for cell attachment, spreading, growth, and differentiation. These include the binding sequence of the amino acid arginine-glycine-aspartic (RGD) and matrix metalloproteinase (MMP)-sensitive degradation sequences. This makes GelMA an ideal platform for controlling cellular behavior and investigating the interactions between cells and materials, as well as for tissue reconstruction^9,10^. GelMA hydrogel is considered an excellent biomaterial that facilitates wound healing in several in vivo models^11–13^.

In recent years, 3D bioprinting has garnered significant attention in the field of tissue engineering as a potential method to fabricate skin substitutes^14,15^. This technique has gained popularity due to its ability to produce and design scaffolds on demand in unique shapes, easily, consistently, and precisely. Several bio-ink hydrogel biomaterials derived from natural sources such as alginate, gelatin, collagen, chitosan, and silk have been reported^16^ and used to create various artificial tissues^17,18^, including bioprinted skin substitutes^19^. Among these biomaterials, GelMA, derived from gelatin, has been recognized as an ideal bio-ink and has become one of the most commonly used materials in 3D bioprinting for tissue engineering research^20^.

Typically, gelatin is primarily sourced from mammalian-based collagens, such as bovine^21,22^ and porcine^23,24^. However, due to cultural and religious constraints associated with these mammalian sources, as well as the risk of infectious disease transmission (e.g., foot-and-mouth disease and bovine spongiform encephalopathy), several alternative natural sources of collagen have been developed to address these issues. These alternatives include marine-derived collagens sourced from materials such as fish scales^25,26^, sea urchins^27,28^, and other marine invertebrates^29^.

Fish-derived gelatin contains amino acids necessary for the human body and the synthesis of GelMA, such as lysine, which reacts with methacrylic anhydride (MA). However, lysine is present in relatively low amounts in the fish gelatin^30,31^, resulting in softer physical properties. These properties provide a more favorable environment for cell viability, cell proliferation, and migration compared to porcine gelatin.

Our recent study, which described the synthesis of GelMA from fish scales, demonstrated that this newly developed GelMA exhibits properties and performance similar to commercial GelMA, making it suitable for use as a bio-ink in 3D bioprinting^26^. However, its low gelatin production yield makes it a less favorable precursor source of GelMA. In addition to fish scales, other fish processing wastes, such as fish skin, could serve as an alternative gelatin source for synthesizing of GelMA^18^. Fish skin-derived GelMA can be combined with other materials, such as alginate, to improve its properties and overcome its limitations. To our knowledge, this previous finding is the first to employ fish skin-derived collagens in the fabrication of 3D GelMA scaffolds^18^. However, the potential of fish skin-derived collagens for the fabrication of 3D GelMA skin substitutes using a 3D bioprinter, as well as its effectiveness in an in vivo study, has not been thoroughly explored.

Since both ASCs have shown promise in promoting skin wound healing^6,13,32^, and human platelet lysate (HPL) have been reported to have beneficial effects on promoting wound healing in several studies^33,34^, the fish skin-based 3D GelMA bio-ink was loaded with ASCs and supplemented with HPL. Herein, we aimed to further explore the potential of using a 3D bioprinter to fabricate a 3D GelMA skin substitutes with fish skin-based GelMA bio-ink in combination with ASCs and supplemented with HPL in a critical-sized full thickness skin defect rat model. The investigation of such a large wound is crucial for advancing clinical treatments, as patient wounds requiring skin grafts are typically extensive. Our findings could provide some important insights into the fabrication and modification of fish-skin-based GelMA hydrogel scaffold as promising material of 3D-printing technology, with potential clinical applications in tissue engineering.

## Experimental methods

### Synthesis of fish skin-based GelMA bio-ink

This method involved a direct reaction between fish skin gelatin (Halamix International Co., Ltd, Thailand) and methacrylic anhydride (MA) (276685, Sigma Aldrich, USA). The fish skin gelatin, which has a viscosity (6.67%w/v at 60 °C) of 3.64 mPa·s and gel strength of 247 g was dissolved in 0.25 M bicarbonate buffer (Ajax Finechem, Australia) to reach a concentration of 10% w/v. The solution was heated with stirring for 30 min at 55 °C until the fish skin gelatin was completely dissolved. Five GelMA solutions were prepared by adding 0.080, 0.085, 0.090, 0.095, and 0.1 mL MA per gram of gelatin, as listed in Table 1. The reactions were maintained for 1 h at 50 °C with a magnetic stirrer and then stopped by adding bicarbonate buffer (5X) and stirring for 20 min at 40 °C. The solutions were dialyzed using a 14 kDa dialysis membrane (Sigma Aldrich, USA) in deionized water at 40 °C for 7 days, followed by centrifugation to remove excess sediment and freeze-drying for 3 days in order to obtain a semi-finished solid GelMA sponge.

**Table 1:** Adding conditions of methacrylic anhydride at various concentrations.

GelMA labels	GelMA conditions (MA/Gelatin)
GelMA 100	0.100 mL/g
GelMA 95	0.095 mL/g
GelMA 90	0.090 mL/g
GelMA 85	0.085 mL/g
GelMA 80	0.080 mL/g

### Preparation of hydrogel

The semi-finished GelMA sponge at different concentrations of 5% and/or 10% (w/v) was dissolved in phosphate-buffered saline (PBS) (Millipore, USA) containing 0.5% 1-[4-(2-hydroxyethoxy)-phenyl]-2-hydroxy-2-methyl-1-propanone (Irgacure 2959, Sigma Aldrich, USA) by stirring for 30 min at 80 °C. The pH of the combined solution was adjusted to between 7.0 and 7.5 with 5 M NaOH. The GelMA solution was sterile filtered (0.2 μm), loaded into a syringe, and stored at 4 °C to increase viscosity. Crosslinking of the GelMA hydrogel was done by exposing it to 365 nm UV light at 10 mW for 3 min before printing onto a petri dish and exposing it to UV light for another 1.5 min. This 3D GelMA sample was used for in vitro and in vivo testing.

### Nuclear magnetic resonance (NMR) characterization and degree of substitution (DS) analysis

^1^H NMR was used to confirm the degree of substitution (DS) of free amino groups on the gelatin by methacrylate groups and to determine the DS of the MA modified gelatin. GelMA was dissolved at room temperature in deuterium oxide (D_2_O 10% w/v) and analyzed using a^1^H NMR spectroscopy (Varian Unity Inova 500 125 MHz). The peak area ratio of modified amino groups to main amino groups was calculated^35,36^ to determine the DS of MA modified gelatin. The DS calculation formula (Eq. 1) is as follows^37^:

1 $${\text{DS}}=S{\prime }/S,$$

where *S’* represents the average integral area of modified amino groups (C = C bond) and *S* represents the average integral area of primary amino groups (–CH–NH bond)^38^.

### Differential scanning calorimetry (DSC) characterization

The thermal properties of the GelMA were investigated using a Rigaku differential scanning calorimeter (Thermo plus EVO2, Rigaku Japan). The scanning was performed from 25 to 100 °C at a rate of 10 °C/min under a nitrogen gas atmosphere.

### Swelling test

The swelling properties of the crosslinked GelMA hydrogel were tested by comparing the weights of the dry and swollen samples using a three-decimal point scale (Mettler Toledo, USA). After weighing, the dried samples were immersed in PBS (Millipore, USA) for 4, 8, 12, 16, 20, and 24 h. The weight of each sample (*n* = 3) was recorded at each time interval. The average weight was used to calculate the percentage swelling ratio using Eq. 2.

2 $${\text{Swelling Ratio }}\left( \% \right){\text{ }}=\frac{{Wt~ - ~W0}}{{W0}}~ \times {\text{1}}00$$

where Wt is the weight of the swelling hydrogel and W_0_ is the weight of the dry hydrogel.

### Mechanical testing

A Universal tensile testing machine (TAXT plus Stable Micro Systems, UK) was used to conduct uniaxial compressive strength tests with a crosshead speed of 0.1 mm/s and a 10 N load cell. A ø10 × 15 mm^3^ GelMA specimen was crosslinked using UV irradiation with a wavelength of 365 nm and an intensity of 20 mW/cm^2^ for 15 min.

### Rheological characterization

Using a TA Instruments rheometer (ARES-G2) (TA Instruments, USA), we performed rheological testing of the fish skin-based GelMA hydrogel (10% w/v) prior to crosslinking. All measurements were performed with a parallel plate geometry (25 mm in diameter). The flow test was performed at 25°C using shear rates of 1 to 500 s^− 1^ to determine viscosity and shear stress. A temperature sweep was conducted from 17 to 29°C with constant shear rates (10 s^− 1^) in order to plot storage modulus (*G’*) and loss modulus (*G”*) values relative to the temperature.

### Cell culture

#### Ethical statement

Human ASCs were harvested via lipoaspiration from the subcutaneous adipose tissue of a patient who had undergone cosmetic surgical procedures. The study protocol (COA. NO. MURA2022/760) was approved by the Human Research Ethics Committee, Faculty of Medicine Ramathibodi Hospital, Mahidol University. Prior to study enrollment, the participants provided written informed consent. We confirm that all experimental protocols were carried out in accordance with relevant guidelines and regulations.

#### Preparation and isolation of human adipose tissue-derived mesenchymal stromal cells (ASCs)

The adipose tissue samples were washed and rinsed with sterile phosphate-buffered saline (PBS) (SH3002803; Hyclone, USA) and finely minced into approximately 0.5 mm^3^ pieces. The minced tissues were enzymatically digested using 0.1% Collagenase Type I (17100017; GIBCO Invitrogen, USA) and then incubated in a shaking water bath at 37 °C for 60 min. The digested tissue solution was then diluted with Dulbecco’s modified Eagle’s Medium (DMEM)-Low Glucose (SH3002102; Hyclone, USA), centrifuged at 1,500 rpm for 10 min and the supernatant was removed. The cell pellets were resuspended with PBS, filtered through a Falcon™ 40 μm cell strainer (352340; Corning, USA), and centrifuged again. The cell pellets were cultured in DMEM complete medium containing 5% human platelet lysate (HPL) (PLTGOLD500GMP; Sartorius, USA), 1% penicillin/streptomycin (15140122; Gibco, USA), 1% 200 mM L-glutamine (25030081; Gibco, USA), and 0.1% amphotericin-B (SV30078.01; Hyclone, USA) at 37 ℃ in a humidified atmosphere of 95% O_2_ and 5% CO_2_, until reaching the third passage.

#### Human ASCs differentiation potential assessment

The ASCs at their third passage were trypsinized and used to determine their trilineage differentiation potential. For adipogenic differentiation, ASCs were plated at a density of 5 × 10^4^ in a T-25 flask and cultured in DMEM complete medium supplemented with 0.035% indomethacin (I7378; Sigma-Aldrich, USA), 0.03% 3-isobutyl-1-methylxanthine (I5879; Sigma-Aldrich, USA), 0.01% dexamethasone (D4902; Sigma, USA), and 0.005% human insulin solution (I9278; Sigma-Aldrich, USA). After 3 weeks in culture, adipogenic-induced ASCs were evaluated by staining intracellular lipid droplets with Oil Red O (O0625; Sigma-Aldrich, USA).

For osteogenic differentiation, ASCs were plated at a density of 5 × 10^4^ in a T-25 flask and cultured in DMEM complete medium supplemented with L-Ascorbic acid (A92902; Sigma-Aldrich, USA), B-glycerophosphate (154804-51-0; Sigma-Aldrich, USA), and 0.001% dexamethasone (Sigma-Aldrich, USA). After 3 weeks in culture, osteogenic-induced ASCs were stained with Alizarin Red S (A5533; Sigma-Aldrich, USA) to evaluate bone matrix mineralization. For chondrogenesis differentiation, ASCs were seeded at a density of 5 × 10^5^ cells per well of a 96 well plate and cultured in StemMACS™ ChondroDiff Medium (130-091-679; Miltenyi Biotec, USA). After 3 weeks in culture, chondrogenic-induced ASCs were stained with Alcian Blue solution (33864-99-2; Sigma-Aldrich, USA) to evaluate aggrecan deposition in cartilage extracellular matrix.

#### ASCs immunophenotypes analysis

Immunotypic mesenchymal cell surface markers on ASCs were characterized by flow cytometry. In brief, ASCs cells (third passage) were washed and resuspended in PBS at a density of 2 × 10^5^ cells per tube and centrifuged at 400 g for 5 min. After centrifugation, the cells were resuspended in 100 µL PBS and incubated with solutions of fluorochrome-conjugated antibodies targeting human CD34, CD45, CD HLA-DR, CD73, CD90, and CD105 (Biolegend, USA) at 4 °C for 30 min in the dark according to the manufacturer’s instructions. Following centrifugation at 400 g for 5 min, cells were washed twice with PBS, resuspended in 500 µL PBS, and analyzed using a flow cytometer (Beckman Coulter, USA).

### Three-dimensional (3D) bioprinting model design and printability

The 3D GelMA hydrogel scaffold was designed with Cellink Heart Ware Repetier – Host Software version 2.1.3 and printed using a 3D bioprinter (CELLINK, Gothenburg, Sweden) in a honeycomb square shape with dimensions of 20 × 20 × 2 mm^3^. The printing conditions were adjusted to obtain continuous extrusion, in which minimum pressure was set to 20–24 kPa with the precision conical nozzle at a diameter size of 30G (inner dimeter 0.233 mm) and the printing nozzle speed of 5 mm/min operating at room temperatures (25 °C). The printability of the fish skin GelMA samples after 8, 15, and 30 min of bio-ink preparation was captured by digital camera to compare the ability to form the designed scaffolds. Before printing, the bio-ink GelMA95 hydrogel was pre-mixed with an aliquot of ASCs (third passage) and was loaded into 3 mL plastic cartridges for printing to obtain a final cell density of 5 × 10^6^ cells per cm^3^ (or per sample). The samples were then crosslinked with ultraviolet light at a wavelength of 365 nm and an intensity of 20 mW/cm^2^ for 3 min.

### Cell viability assay

Cell viability of the ASCs in the 3D GelMA samples was accessed using PrestoBlue (A13261; Thermo Fisher, USA) according to the manufacturer’s instructions. In brief, the 3D GelMA hydrogel scaffolds and ASC-loaded 3D GelMA hydrogel scaffolds were cultured in a 6-well cell culture dish (Corning, USA) containing Dulbecco’s modified Eagle’s medium (DMEM) supplemented with 10% fetal calf serum (Life Technologies-Gibco) and 100 mg/mL Penicillin–Streptomycin (Life Technologies-Gibco) for 24, 72, and 120 h. The samples were then washed twice with DMEM complete medium and incubated (Forma Scientific, USA) in the dark for 1.5 h in fresh culture medium (1.8 mL) and PrestoBlue (200 µL). The medium solution was collected and placed in a 96-well plate (Corning, USA) in triplicate (100 µL/well/sample). The optical density at a wavelength of 570 nm was used to evaluate cell viability over time using a microplate reader (model Infinite 200 Pro; Tecan, Switzerland). Cell viability was calculated using Eq. 3.

3 $${\text{Cell viability}}\left( \% \right)=\frac{{O.D.~~of~treatment}}{{O.D.~~of~control}} \times {\text{1}}00$$

where O.D. of treatment refers to the absorbance values of the sample group and O.D. of control refers to the absorbance values of the control group.

### In vivo study design

#### Animals and ethics statement

All surgical and experimental procedures used in this study were approved by the Institutional Animal Care and Use Committee (IACUC) of the Faculty of Science, Mahidol University (Protocol No. MUSC65-008-601) and carried out in accordance with the ARRIVE guidelines (https://arriveguidelines.org) and other relevant guidelines and regulations.

#### Experimental groups

Twenty-seven adult male Wistar rats (8–9 weeks old, 250–280 g) obtained from Nomura Siam International Co., Ltd., Bangkok, Thailand, were acclimatized for at least 5 days before commencing any experimentation and were housed in an Association for the Assessment and Accreditation of Laboratory Animal Care (AAALAC) accredited facility with a 12-h dark/light cycle, 22 ± 1 ˚C, and 30–70% humidity. Standard rat chow and water were supplied *ad libitum*. Rats were randomized to three groups (*n* = 9/group): Group-I: untreated group (wound without treatment), Group-II: GelMA group (wound transplanted with GelMA alone), and Group III: GelMA + ASCs + HPL (wound transplanted with ASCs-loaded 3D GelMA supplemented with HPL).

#### Critical-sized full-thickness skin defect rat model

**Fig. 1:**
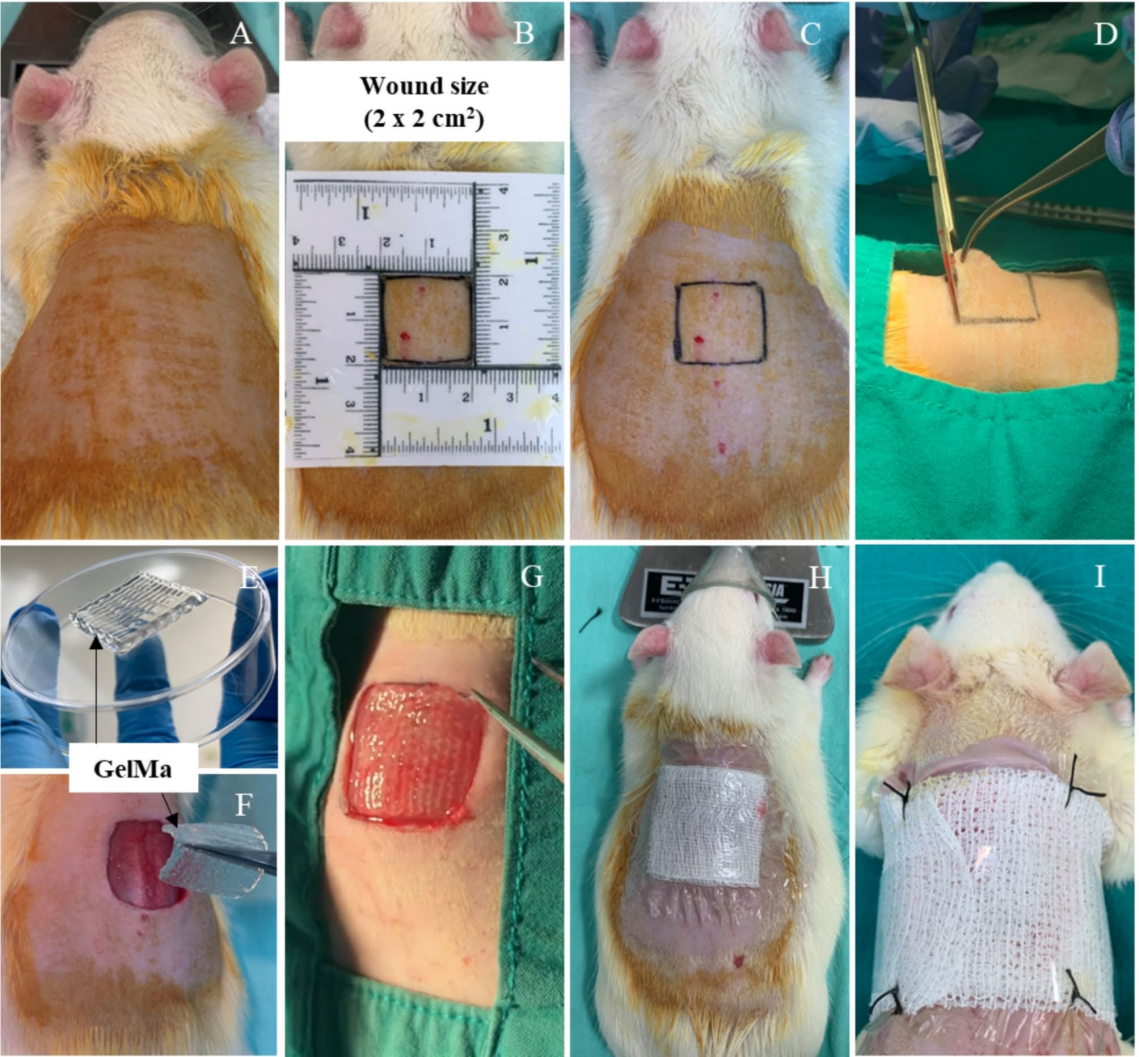
Critical-sized full-thickness skin defect procedure and 3D GelMA hydrogel scaffold transplantation. (**A-C**) Under anesthesia, the dorsal mid-lumbar region of the rat was shaved and cleaned, and the defect size was marked. (**D**) A critical-sized (2 × 2 cm^2^) square-shape piece of skin was excised, and (**E-G**) the defect was transplanted with either GelMA alone or GelMA + ASCs + HPL. (H) The wound was covered with BACTIGRAS antiseptic dressing, gauze, and Tegaderm transparent film dressing. (I) To secure the transplant in place, four stitches attaching the bandage to the skin were applied.

Rats were anesthetized by inhalation of isoflurane (Attane™, Piramal Critical Care. Inc., USA) in oxygen (5% for induction; 2–3% for maintenance). Hair on the dorsal mid-lumbar region of each rat was removed using a razor blade, and the surgical area on the skin was cleaned with 70% ethanol followed by povidone iodine solution (Fig. 1A). Subsequently, a 2 × 2 cm^2^ square was marked using square ruler template and marking pen (Fig. 1B-C). The square-shape piece of skin was excised using scalped blade No.21, dissecting scissors, and forceps (Fig. 1D).

For groups II and III, either 3D GelMA alone or 3D GelMA + ASCs + HPL was transplanted onto each wound with the help of forceps (Fig. 1E-G). The wound was left untreated in group I. After transplantation, each wound was covered with BACTIGRAS antiseptic dressing (2.5 × 2.5 cm), gauze (2.5 × 2.5 cm), Tegaderm transparent film dressing (10 × 12 cm), and wrapped loosely with an elastic cohesive bandage to prevent the 3D GelMA sample from slipping off of the defect (Fig. 1H). Four stitches were used to attach the bandage to the skin (Fig. 1I). Each rat was given tramadol (10 mg/kg; Tramada-100, L.B.S. Laboratory Ltd. Part., Bangkok, Thailand) once daily for 3 days and cefazolin (20 mg/kg; Cefaben; L.B.S laboratory Ltd, Part., Bangkok, Thailand) once daily for 5 consecutive days via subcutaneous injection to provide pain relief and prevent post-operative infection, respectively.

#### Assessment of the unclosed wound area (%)

To assess the percentage of unclosed wound area, a small ruler was placed close to the wound defect area as a reference scale. The wounds were photographed at 0 (immediately after wound creation), 3, 7, 10, and 14 days post-wound creation using an iPhone XR (Apple, USA). The wound images were analyzed using ImageJ software. The unclosed (remaining) wound area (%) post-wound creation was calculated using Eq. 4.

4 $${\text{Unclosed wound area }}\left( \% \right)=\frac{{A0~ - ~At}}{{A0}} \times {\text{1}}00$$

where A0 is the initial wound area and At is the remaining wound area at different time points (i.e., 3, 7, 10, and 14 days post-wound creation).

#### Histological and immunohistochemical (IHC) evaluation

On day 14 after wound creation, rats were euthanized via intraperitoneal injection of an overdose of thiopental (100 mg/kg; Anesthal, Scott-Edil Pharmacia Ltd., Solan, India) and the full-thickness wound areas were harvested. The tissue samples were fixed in 10% neutral buffered formalin for 48 h and processed with routine histological tissue processing and paraffin embedding, followed by sagittal sectioning at 4 μm thickness. Tissue sections were then stained with hematoxylin and eosin (H & E) and Masson’s trichrome (MT) according to the manufacturer’s recommendations to visualize general tissue morphology and observe collagen deposition in the full-thickness wound.

IHC staining for type I collagen and CD31 was performed using an anti-collagen I antibody (1: 500, ab254113, Abcam, Cambridge, MA) and an anti-CD-31 antibody (1:500, SC-376764, Santa Cruz Biotechnology, USA), respectively. Briefly, tissue sections were dehydrated and processed for antigen retrieval by incubating them in 10mM sodium citrate buffer at 60° C for 30 min. Endogenous peroxidase was blocked with 3% H_2_O_2_ for 10 min. Nonspecific binding was prevented by incubating sections in 1% normal goat serum in PBS at 37° C for 30 min. Next, sections were incubated overnight at 4° C with anti-collagen type I and anti-CD-31 antibodies. After washing with 1X PBS, sections were incubated with biotinylated goat anti-rabbit IgG (ab64256, Abcam) and HRP-conjugated Streptavidin (ab64269, Abcam) according to the manufacturer’s instructions. Diaminobenzidine (DAB) reagent (ab64238, Abcam) was used for visualization, followed by hematoxylin for nuclei staining. Slides were examined using a light microscope (Nikon Eclipse Ts2R microscope) equipped with a Nikon DS-Fi3 camera, and images were captured using NIS-Elements D version 5.21.00 software.

#### Ex vivo evaluation of collagen regeneration

The ex vivo analysis of collagen regeneration during wound healing was evaluated using second harmonic generation (SHG) and SP8 DIVE multiphoton microscopy (Leica Microsystems, Germany) equipped with an Insight X3 Laser for two-photon excitation and Leica 4 Tune spectral hybrid detectors for signal collection. This SHG technique offered a non-invasive method for observing collagen formation around the edge of the wound areas at day 14 post-wound creation (the study endpoint). The representative SHG signal intensity of collagen was captured from biopsy samples (one sample per group) using an HC PL IRAPO 25x/1.00 WATER objective lens with a 1.0 NA. The excitation wavelength was set at 900 nm. Laser power was set at 2.5 W, and image gain was fixed at 100 during the acquisition period. Images were acquired in 24-bit with a 1024 × 1024-pixel dimension. A motorized stage was used to acquire a series of images at tiled x/y locations. Individual tiles were acquired and stitched using Leica LAS X software (version 3.5.6.21594).

### Statistical analysis

Statistical analyses were performed using GraphPad Prism version 9.0.0 (GraphPad Software, La Jolla, CA, USA). Data are expressed as mean ± standard error of the mean (SEM) unless otherwise indicated. The distribution of data was tested using the Shapiro-Wilk test. For normally distributed data, multiple sets of data, including swelling ratio (%), compressive strength (kPa), cell viability (%), and unclosed wound area (%), were performed using the two-way analysis of variance (2-way ANOVA), followed by the Bonferroni multiple comparison test. Non-normally distributed data, including histopathological scoring data, were analyzed using the Kruskal-Wallis test and Dunn’s multiple comparisons test. The statistical significance criterion was *p* < 0.05.

## Results and discussion

### Fish skin GelMA synthesis and degree of substitution

**Fig. 2:**
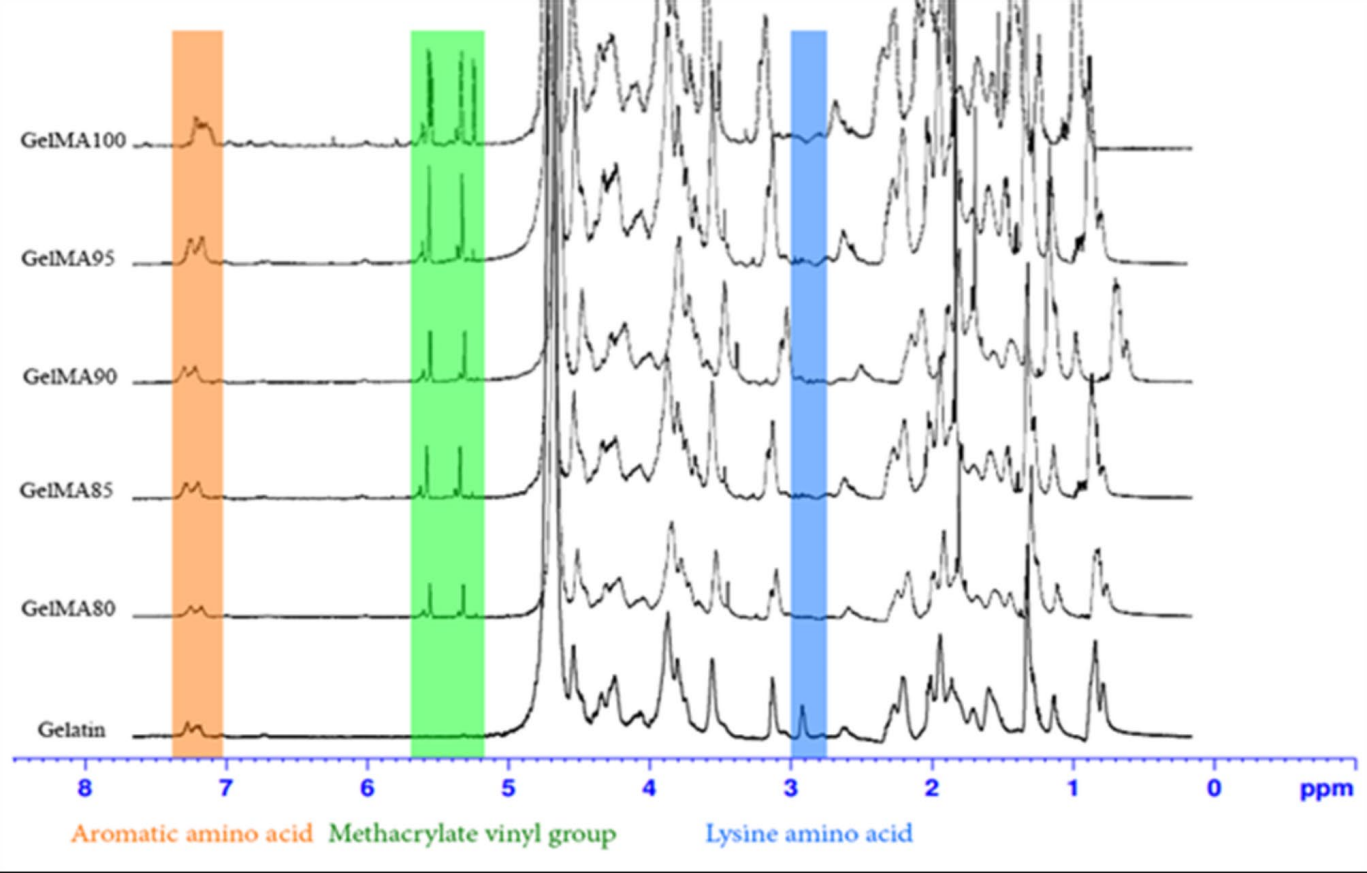
^1^H-NMR spectra of obtained GelMA samples compared to fish skin gelatin.

The fish skin gelatin sample was reacted with methacrylic anhydride (MA) at a feed ratio of 0.5 mL/min for 1 h, similar to previous reports^39^. As shown in Fig. 2, NMR characterization verified that GelMA was synthesized from the constituent gelatin and MA^35^. The reaction of fish skin gelatin with MA resulted in methacrylamide group replacement, indicated by additional peaks around 5.3 and 5.7 ppm, which were attributed to the acrylic protons of methacryloylated grafts and the methacrylate vinyl group clings (Fig. 2). Additionally, the peak for lysine methylene identified at 2.8–3.0 ppm in pure gelatin steadily diminished with increasing degrees of methacrylamide group replacement. A low integration value indicates that the fish skin gelatin interacts ultimately with all MA molecules, resulting in enhanced methacryloylation of lysine amino acid groups.

From Eq. 1, the degree of substitution (DS) was calculated by comparing the proton signal at δ = 2.8–3.0 ppm (the protons of methylene of lysine signal) and GelMA signal at δ = 5.3–5.7 ppm (the protons of methacrylate vinyl group of MA). The DS of the fish skin gelatin sample produced in this study was 93.46%, indicating a similar degree of substitution compared to porcine and bovine skin GelMA (94%), as shown in a previous report^40^.

The spectrum of fish skin gelatin, modified by adding different amounts of methacrylic acid to each of the five samples, showed specific peaks. In the range of 5 to 6 ppm and at 1.8 ppm, the spectrum indicated the presence of C = O bond groups or vinyl methacrylate groups (methacrylate vinyl group). A peak around 2.9 ppm indicated the presence of N–H groups or lysine amino acid groups. The spectral range of lysine was lower than that of unmodified gelatin, suggesting a reaction with the acid and complete modification by methacrylic anhydride. The results from all 5 samples showed the level of substitution of the methacrylamide group, obtained by integrating the area under the remaining lysine curve of methacryloyl gelatin. The specific peak area was compared with the area under the lysine curve of the precursor gelatin using the TopSpin program. When calculated according to Eq. 1, the degrees of methacrylate group substitution were 78.07%, 82.40%, 86.98%, 91.43%, and 92.19%, respectively, as shown in Table 2.

**Table 2:** Degree of substitution of fish skin GelMA samples.

Formulas	DS = 1- S’/S * 100	Results
100	DS = 1- 0.0564/0.7228 *100	92.19%
95	DS = 1- 0.0619/0.7228 *100	91.43%
90	DS = 1- 0.0941/0.7228 *100	86.92%
85	DS = 1- 0.1272/0.7228 *100	82.40%
80	DS = 1- 0.1585/0.7228 *100	78.07%

### Thermal properties of Fish skin GelMA

**Fig. 3:**
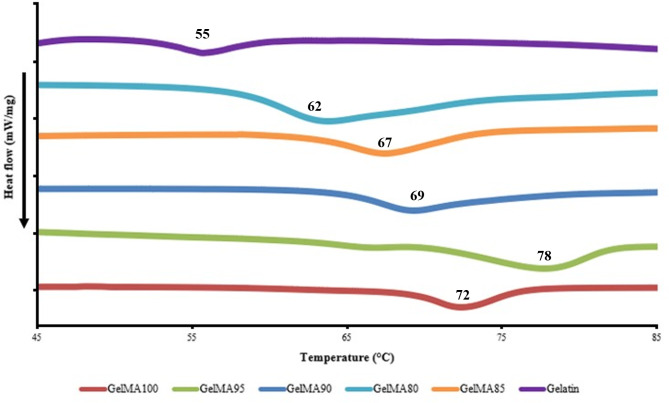
DSC thermogram of the fish skin GelMA samples.

The thermal property analysis of the fish skin gelatin and five GelMA samples showed a broad endothermic peak, or melting temperature, at approximately 55 °C for the original fish skin gelatin. The five GelMA samples had melting temperatures of approximately 62, 67, 69, 78, and 72 °C, as shown in Fig. 3.

The melting temperatures correlated with the proportion of MA used in the synthesis, indicating that the level of methacryloyl group substitution proportionally affects the thermal properties of the synthesized samples. However, GelMA95 had a higher melting point than GelMA100, which suggests the evaporation of remaining water. According to Velasco Rodriguez et al., this may be due to lower water availability and/or more tightly bound water molecules to the gelatin methacryloyl chain^41^.

### Swelling properties of the fish skin GelMA samples

**Fig. 4:**
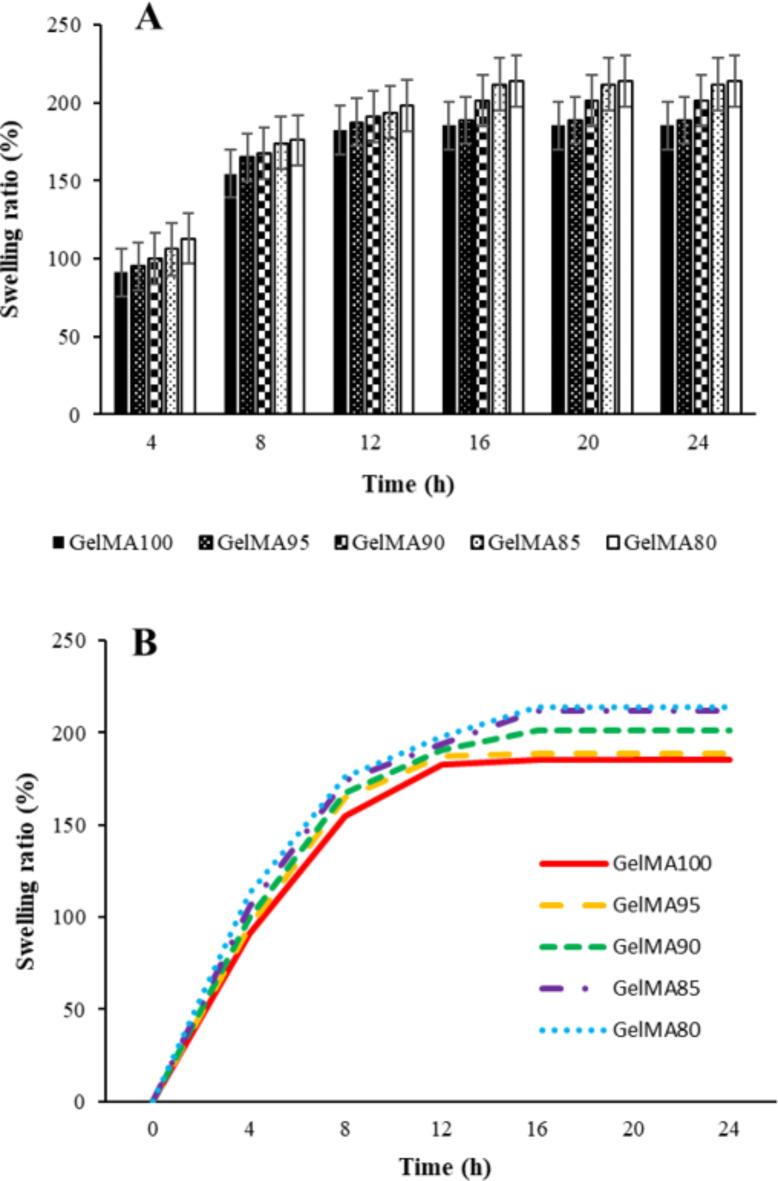
(**A**) Bar graph and (**B**) trend curve showing the swelling rate of the fish skin GelMA samples over a 24-h period. Data are expressed as mean ± SD (*n* = 3/sample).

Testing the swelling properties of the five gelatin methacryloyl samples with different levels of methacryloyl group substitution at a concentration of 10% in PBS revealed the following results (Fig. 4). After crosslinking each sample for 15 min and immersing them in PBS for 24 h, GelMA80 had the highest swelling rate at 213.89%, while GelMA100 had the lowest at 185.44%. The concentration of methacrylic anhydride was inversely proportional to the swelling ratio of GelMA, as illustrated in the bar graph showing the percentage swelling rates of the five samples over 24 h (Fig. 4A).

As shown in Fig. 4B, the duration of soaking in PBS was directly proportional to the swelling ratio until it reached saturation at about 200% after 12 h. Previous research has reported that the swelling ratio of GelMA is affected by the level of methacryloyl group substitution and the concentration of MA added to the fish skin gelatin^42^. A lower degree of substitution or a lower concentration of MA increases the swelling ratio^43^. For hydrogel application, a saturated bio-ink that maintains a low swelling rate allows for better control of size and shape compared to a high-swelling bio-ink. In Fig. 4B, the swelling ratios of GelMA95 and GelMA100 were identical at about 185% after the 12-h saturation period.

For 3D-printed skin substitute fabrication, the bio-ink hydrogel should have a high melting temperature, a high DS, and a low saturated swelling ratio. Considering the combination of DS (GelMA95 is comparable to GelMA100 at 91–92%), melting temperature (the highest for GelMA95 at 78 °C), and similar swelling properties, it can be concluded that GelMA95 is most suitable for further investigation.

### Mechanical properties and printability of fish skin GelMA

**Fig. 5:**
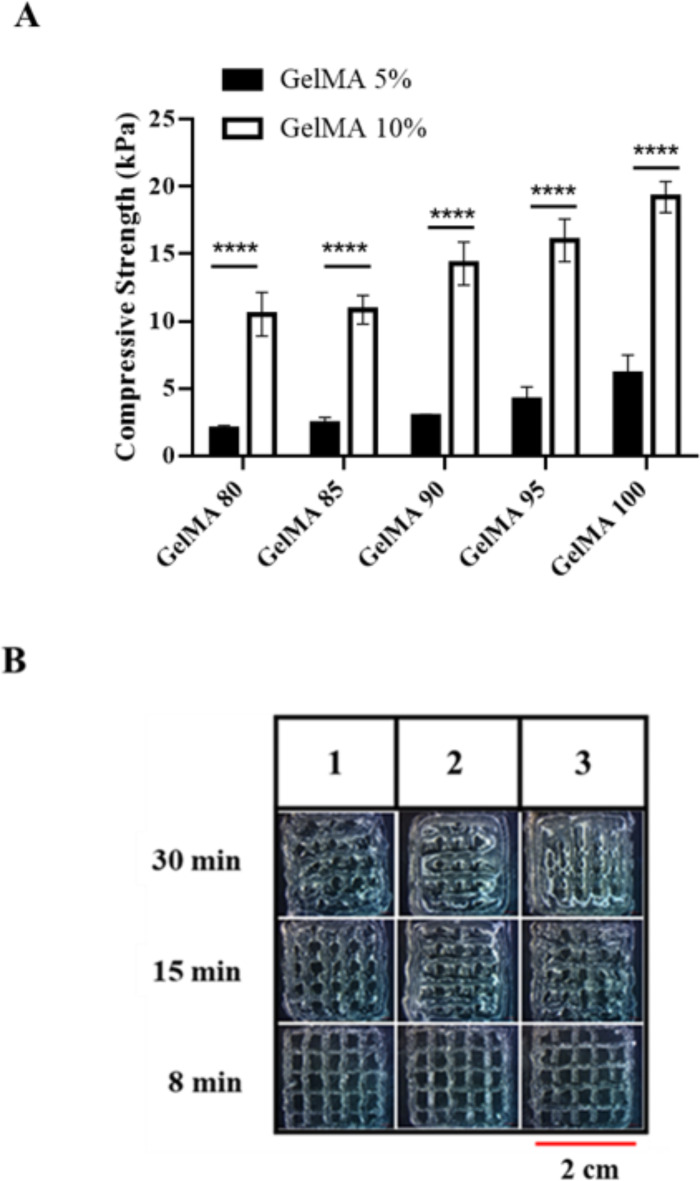
(**A**) A compressive strength test of the fish skin GelMA samples (*n* = 3/sample). Data are expressed as mean ± SD. Statistically significant differences were assessed between the two experimental groups as indicated. (**B**) Printability of the fish skin GelMA95 sample at a concentration of 10% of PBS (w/v) after 8, 15, and 30 min of bio-ink preparation.

As shown in Fig. 5A, the compressive strength of the five GelMA samples was tested at concentrations of 5 and 10% in PBS. After crosslinking each sample for 15 min and then immersing all samples in PBS for 24 h, GelMA100, at a concentration of 10%, showed the highest compressive strength, and GelMA80, at a concentration of 5%, showed the lowest compressive strength.

At a concentration of 10% in PBS (w/v), GelMA95 had the best ability to form within 8 min after ink preparation (Fig. 5B). Over time, the ability to form a scaffold gradually decreased until the hydrogel could no longer form a shape. These results suggest that the prepared hydrogel may require immediate printing due to its rheological properties as a thermal gel with a low gelling point. As time elapses, the viscosity of the hydrogel decreases, impacting printing efficiency. Therefore, the printing process must consider the duration which the gel maintains its viscosity to ensure optimal printing efficiency.

Quantifying printability is challenging due to the irregular and inconsistent nature of the printed samples. As shown in Fig. 5B, GelMA exhibits optimal printability at 8 min after removal from the refrigerator (about 4 °C). At 15 min, the increasing temperature causes GelMA to become less viscous, reducing print quality. By 30 min, GelMA becomes even more liquid, resulting in further reduction of print quality. The main difference lies in the line characteristics, which change due to the increasing temperature of GelMA. This causes the extruded ink to have larger dimensions, and in some areas, it flows and merges, deviating from the original design. This inconsistency makes it inappropriate to report the data quantitatively. Consistent with previous findings, most studies presented such data qualitatively or as a phase diagram with markers^44,45^. Based on our study, we relied on rheology results to determine the optimal printing range and time the samples after removing then from the refrigeration.

GelMA 95 exhibited the highest compressive strength of 14.28 kPa at a concentration of 10%. At a concentration of 5%, the compressive strength was 2.98 kPa, as shown in Table 3. Previous research has shown that the level of substitution of the methacryloyl group affects the compressive strength of the MA-modified fish skin gelatin. As the degree of substitution of the methacryloyl group increases^42^ or the concentration of gelatin methacryloyl increases, the compressive strength will also increase^43^. Additionally, the completeness of crosslinking can influence compressive strength. Increasing the amount of crosslinking agent or the duration of exposure to UV light at 365 nm enhances the completeness of crosslinking, thereby affecting the strength of the hydrogel scaffold and increasing the compressive strength of GelMA^46^.

**Table 3:** Compressive strength and ultimate strain of fish skin GelMA95.

Concentration (%w/v)	Compressive Strength (kPa) (*n* = 3)	Ultimate Strain (*n* = 3)
5	2.93	0.44
10	14.28	0.41

### Rheological properties of Fish skin GelMA

**Fig. 6:**
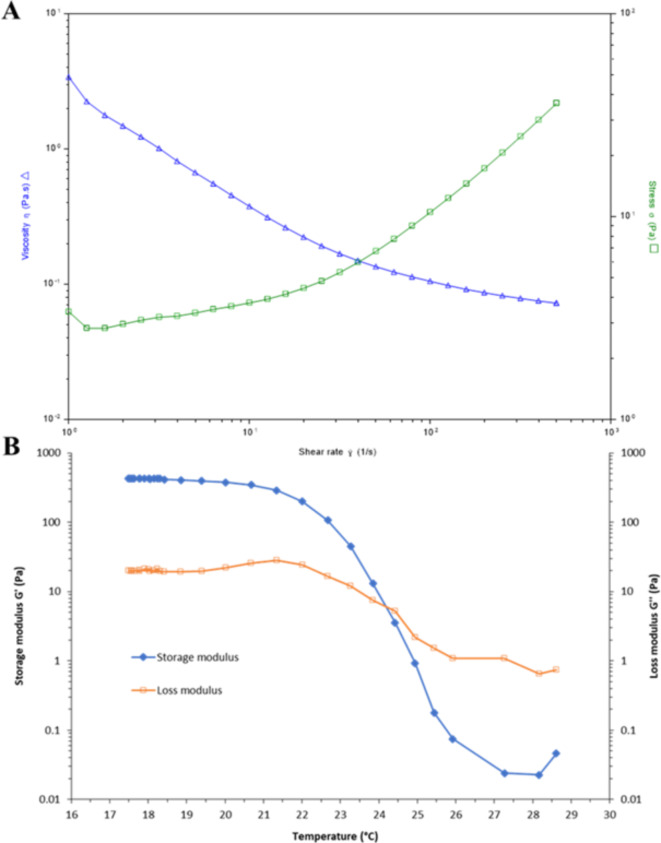
(**A**) Viscosity and shear stress profiles relative to shear rate of the 10% w/v fish skin GelMA solution at 25 °C, (**B**) Temperature sweep test of the 10% w/v fish skin GelMA solution.

The effect of shear rate on the shear stress and viscosity of fish skin GelMA hydrogel 10% w/v before crosslinking was examined using a rheological flow test at room temperature (25 °C). As the shear rate increased, the viscosity decreased, and shear stress increased (Fig. 6A), demonstrating that shear stress decreased the viscosity of the fish skin GelMA. This type of behavior is classified as non-Newtonian or pseudo-plastic with shear thinning^47^. We demonstrated that pushing the fish skin GelMA 10% w/v solution through a syringe can overcome the lowered viscosity and allow it to flow effectively.

The temperature sweep test of the fish skin GelMA hydrogel assessed its rheological properties at a constant shear rate of 10 s^− 1^ to determine optimal conditions for syringe application. Figure 6B shows the shear stress and temperature sweep viscosity profiles of the 10% w/v fish skin GelMA solution before crosslinking. The storage modulus (*G’*) began decreasing at 20°C and crossed the loss modulus (*G”*) curve at 24 °C. This indicates that the fish skin GelMA material transitions from a gel (semi-solid) to a liquid state between 20 and 24 °C, accompanied by reduced viscosity. It may be inferred that at temperatures below 20 °C, fish skin GelMA solution remains viscous enough for extrusion and syringe application without becoming too runny. UV crosslinking can then further stabilize the GelMA material for adherence to the skin wound^39^.

### Cell viability assessment

Human primary ASC culture was characterized by general cell morphology and specific markers of mesenchymal cell lineages. Consistent with our previous and other findings^48,49^, the ASCs appeared as a heterogeneous population of flattened, elongated, fibroblastic-like, and spindle-shaped cells (See Supplemental Fig. 1). We confirmed the presence of MSCs by the positive expression of CD73, CD90, and CD105, and the negative expression of CD34, CD45, and HLA-DR using flow cytometry (See Supplemental Fig. 2). The ASCs were able to differentiate into adipocytes, osteocytes, and chondrocytes, confirming their trilineage differentiation potential (See Supplemental Fig. 3).

**Fig. 7:**
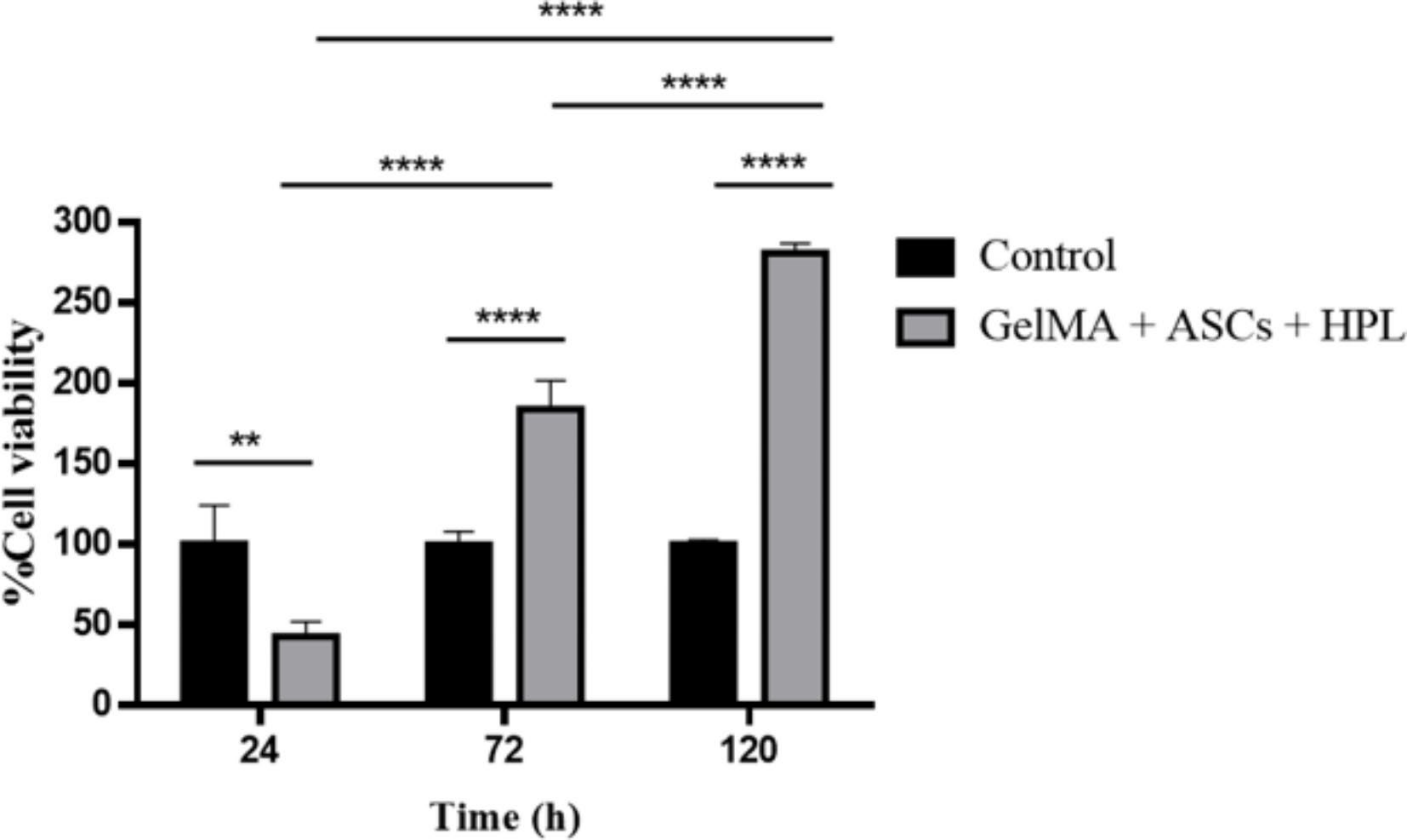
The cell viability (%) of ASCs in the 3D GelMA hydrogel scaffold over 120 h. Data are expressed as mean ± SD. Statistical differences were assessed between the two experimental groups at the same time-point as indicated.

The viability of ASC cells in the 3D GelMA hydrogel scaffold were compared to a standard cell culture control group with PrestoBlue over a culture period of up to 120 h (Fig. 7). After 24 h in culture, we determined the cell viability of ASCs within the cell scaffolds to be 42.9 ± 9.1%. It is considered very low, which could be due to the duration of the proliferation experiment. Both the formation and crosslinking may affect the cell viability^50^. Over time, the viability of the ASCs within the cell scaffolds increased to 184.0 ± 17.9% and 281.2 ± 5.9% at 72 and 120 h, respectively, which may be attributed to the increased number of cells within the 3D scaffold^51^. In addition, HPL may also attribute to this result as it has been demonstrated previously that it could improve human ASCs cell proliferation, colony forming unit and reduce cell senescence^52^.

In comparison, the viability of the control group did not increase over time, likely due to the limitations of cell growth on the 2D surface of a culture dish^53^. Our results are consistent with the previous research by Athirasala et al.^54^, which showed that the number of cells increased significantly within the 3D GelMA hydrogel scaffold.

### In vivo assessment

**Fig. 8:**
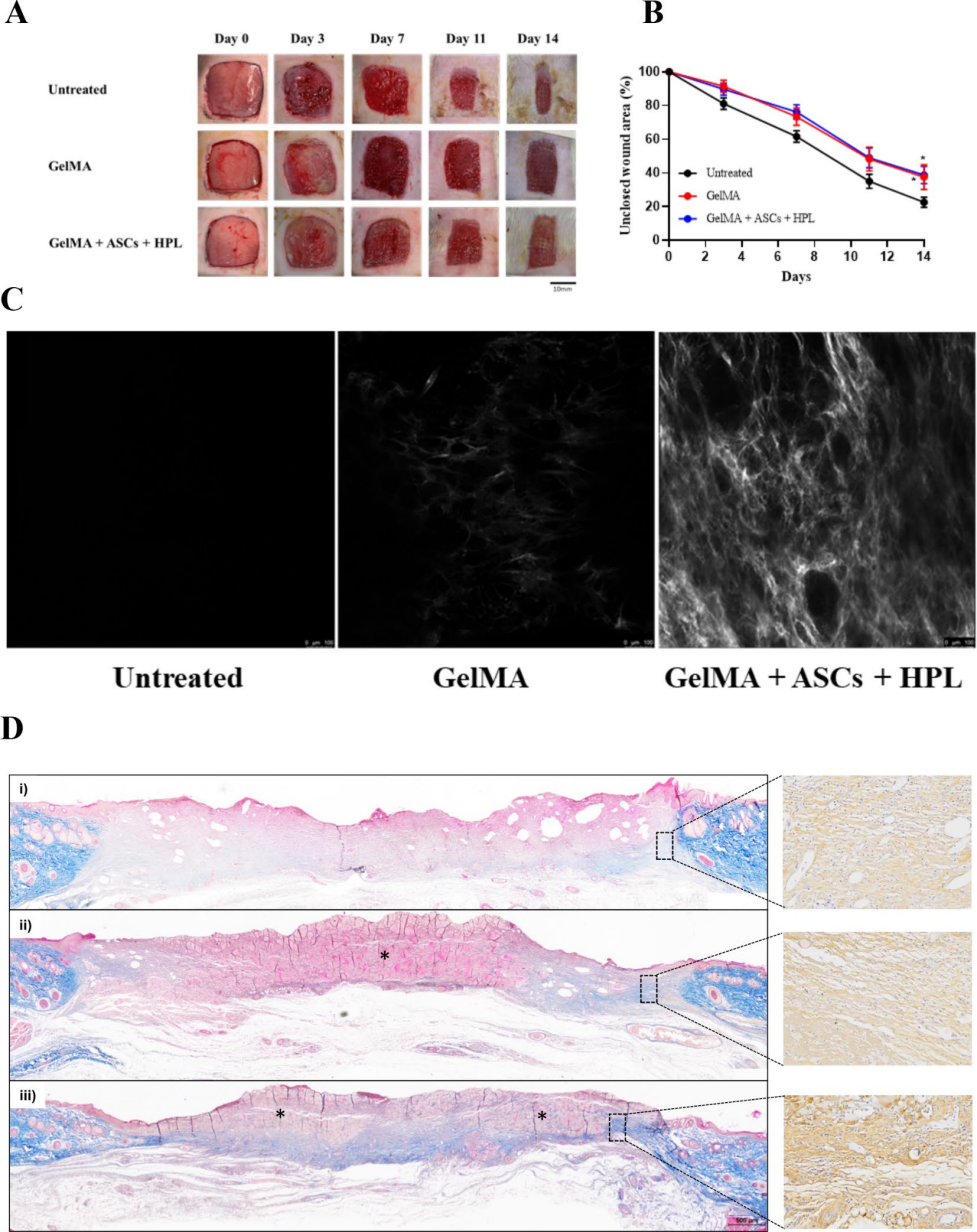
(**A**) Photographs of square wound closure kinetics at day 0, 3, 7, 10, and 14 during the wound healing process of three experimental groups: untreated, GelMA, and GelMA + ASCs + HPL. (**B**) The percentage of unclosed wound area for each experimental group; data are expressed as mean ± standard error of the mean (*n* = 9/ group). (**C**) Representative SHG gray scale intensity images of collagen deposition from biopsy samples taken at day 14 post-wound creation. (**D**) Representative histological images of Masson’s trichrome (MT) staining of wound Sect. (40×): (i) untreated wound, wound treated with either (ii) GelMA or (iii) GelMA + ASCs + HPL at day 14. Blue staining indicates collagen fiber formation. GelMA + ASCs + HPL demonstrated deep blue staining compared with the other groups (untreated wound and wound treated with GelMA). Higher magnification (200 ×) of immunohistochemistry staining for type I collagen enlarged from the black dotted square of each correspondence image. Scale bars are 100 μm in (**C**) and 500 μm in (**D**).

The effectiveness of the 3D bioprinted fish skin GelMA loaded with ASCs and HPL was assessed in a critical-sized full-thickness skin defect rat model. Figure 8A shows images of the wound over 14 days after wound creation. The wound sizes in all experimental groups gradually decreased over time, as shown by wound closure kinetics (Fig. 8B). Quantitative image analysis revealed that the percentage of unclosed wound area in the untreated group (22.6 ± 3.0%) was significantly smaller than that of the GelMA (37.5 ± 7.4%; *p* < 0.0333) and GelMA + ASCs + HPL groups (38.8 ± 5.0%; *p* < 0.0194) at day 14 post-wound creation (Fig. 8B). Interestingly, the untreated wound appeared to close earlier than the wounds treated with GelMA and GelMA + ASCs + HPL. Our results align with a previous study reporting that GelMA degrades slowly in vivo (approximately 4 weeks)^55^. Therefore, it is plausible that the remaining GelMA observed in our study may have prevented or delayed wound closure. It has been suggested that the GelMA bio-ink could maintain a moist wound environment, which offers several benefits, including promoting the wound-healing process by preventing dehydration and enhancing angiogenesis, epithelization, and collagen synthesis^14^. The delayed wound closure may provide a beneficial impact as it could help avoid complications of massive wound healing, such as extensive scarring and wound tension^56^. In addition, histological findings did not reveal any immunogenic response (i.e., no necrosis, hemorrhage or extensive foreign body reaction) in any of the experimental groups, regardless of the implanted samples. This is consistent with previous studies that demonstrated collagen extracted from fish skin does not induce any immune responses^57–60^.

In this study, we further demonstrate the formation of collagen fibers in situ without additional processing and staining by using the SHG technique. For each treatment, three representative samples from skin biopsies were selected on day 14 after wound creation. We found that wound samples transplanted with GelMA + ASCs + HPL exhibited marked increases in SHG intensity at the wound edges, indicating the formation of new collagen fibers (Fig. 8C). In contrast, minimal SHG signals were observed in samples transplanted with GelMA alone or in those left untreated. Therefore, we postulated that the addition of ASCs and HPL to GelMA is necessary to promote wound healing, potentially through the paracrine secretion effect from the ASCs^61^, which enhances collagen deposition and angiogenesis during the granulation tissue formation phase of the wound healing process. This finding is aligned with the previous studies, which reported that gelatin-based hydrogel loaded with ASCs facilitates full-thickness skin wound healing^6,13^.

In the skin, collagen plays an essential role in regulating the wound-healing process. Type I and III collagen are synthesized during wound healing and maturation. Type III collagen synthesis predominates during the acute phase of normal wound healing (inflammatory and early proliferative phases), whereas type I collagen synthesis occurs in the late stage (proliferative and maturation phases) of the healing process^62,63^. In the present study, Masson’s Trichrome and IHC staining demonstrated greater type I collagen formation in wounds treated with GelMA + ASCs + HPL compared with untreated wounds and wounds treated with GelMA alone (Fig. 8D). Although we did not quantify the staining in this preliminary study, using the SHG technique, our histological findings confirm the formation of collagen fibers in situ. In the present study, we focused on the proliferative and maturation phases of wound healing, in which type I collagen is predominant; thus, evaluation of type III collagen was not performed. The increase of type I collagen in the wound transplanted with GelMA + ASCs + HPL, compared to the untreated wound and wound treated with GelMA alone, suggests that GelMA loaded with ASC and HPL may induce type I collagen synthesis to facilitate the wound healing process, especially in the late phase.

**Fig. 9:**
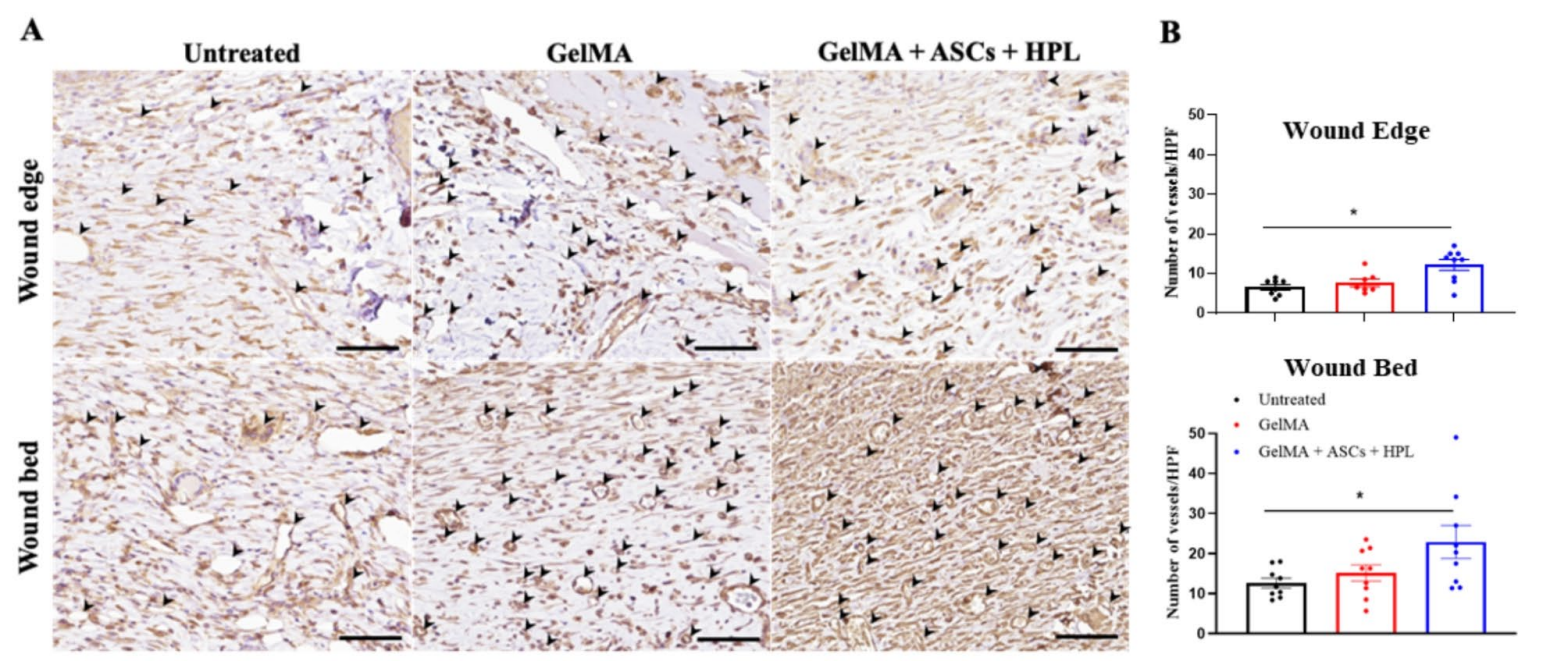
(**A**) Histological images of wound sections stained with anti-CD31 for the untreated wound and wounds treated with GelMA or GelMA + ASCs + HPL at day 14. New blood vessel formation (neovascularization) is indicated by arrowheads. Bar = 50 μm. (**B**) The number of blood vessels on day 14 of the wound treated with GelMA + ASCs + HPL was significantly higher than the untreated wound at the wound edge and wound bed.

In vivo angiogenesis was also evaluated by immunostaining for CD31, a surface marker of vascular endothelial cells widely used as a marker of angiogenesis^13,64^. A significant increase in the number of blood vessels in the wound site was observed in both the wound edge and wound bed of wounds treated with GelMA + ASCs + HPL at day 14 post-wound creation (Fig. 9A). The semi-quantitative analysis of new blood vessels is shown in Fig. 9B. It has been suggested that GelMA, especially when combined with pro-angiogenic agents, enhances vascularization, thereby promoting wound healing^65^. It is known that human blood-derived factors, including platelet lysate, enhance angiogenesis and promote cell migration in the normal wound healing process. For example, human platelet lysate (HPL) and platelet-rich plasma (PRP) contain many pro-angiogenic components (e.g., PDGF, FGF-2, VEGF-A, EGF, platelet factor-4), which have been shown to improve neovascularization and wound healing^66^. Consistent with this, our study demonstrated the highest vessel formation in the wounds that were treated with GelMA + ASCs + HPL compared to those treated with GelMA alone and untreated wounds.

## Conclusions

In summary, we successfully synthesized and developed fish skin-based GelMA bio-inks with mechanical properties suitable for 3D bioprinting. These bio-inks can be combined with ASCs and HPL using a 3D bioprinting technique to create a fish skin-based GelMA skin substitute. This 3D GelMA scaffold demonstrated excellent biocompatibility with ASCs, effectively promoting cell viability and proliferation over a culture period of up to 120 h in vitro. The efficacy of this 3D fish skin-based GelMA scaffold material was further confirmed in a critical-sized full-thickness skin defect rat model, possessing good biocompatibility, supporting collagen deposition, and promoting angiogenesis without causing immunological rejection. Taken together, the 3D ASCs + HPL-loaded GelMA scaffolds present significant therapeutic potential as a skin substitute.

## Electronic supplementary material

Below is the link to the electronic supplementary material.

Supplementary Material 1

## Data Availability

The data presented in this study are available on request from the corresponding author.
