# Supplementary material for: 3D bioprinting of fish skin-based gelatin methacryloyl (GelMA) bio-ink for use as a potential skin substitute
Source: Sci Rep. 2024 Oct 5;14:23240. doi: 10.1038/s41598-024-73774-1 (PMC11455937; doi:10.1038/s41598-024-73774-1)
Supplement: Supplementary file 1 — Supplementary Material 1 [file 41598_2024_73774_MOESM1_ESM.docx]

**Supplementary information**


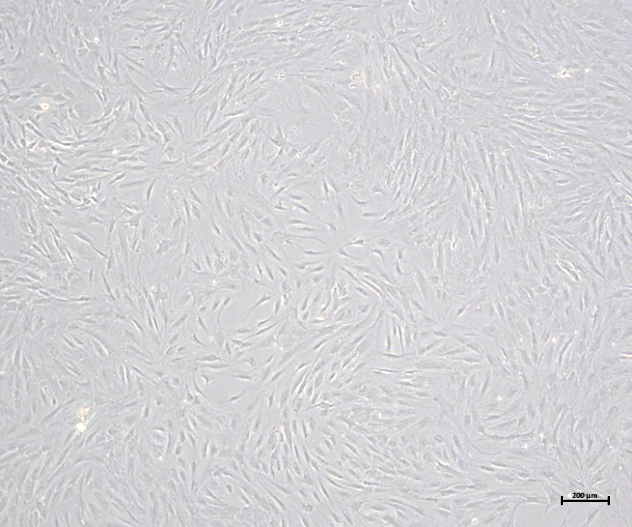


**Supplemental Figure 1** The morphology of human adipose tissue-derived mesenchymal stem cells (Passage 3) at day 7 under a standard light microscopy. Scale bar = 200 µm.

**
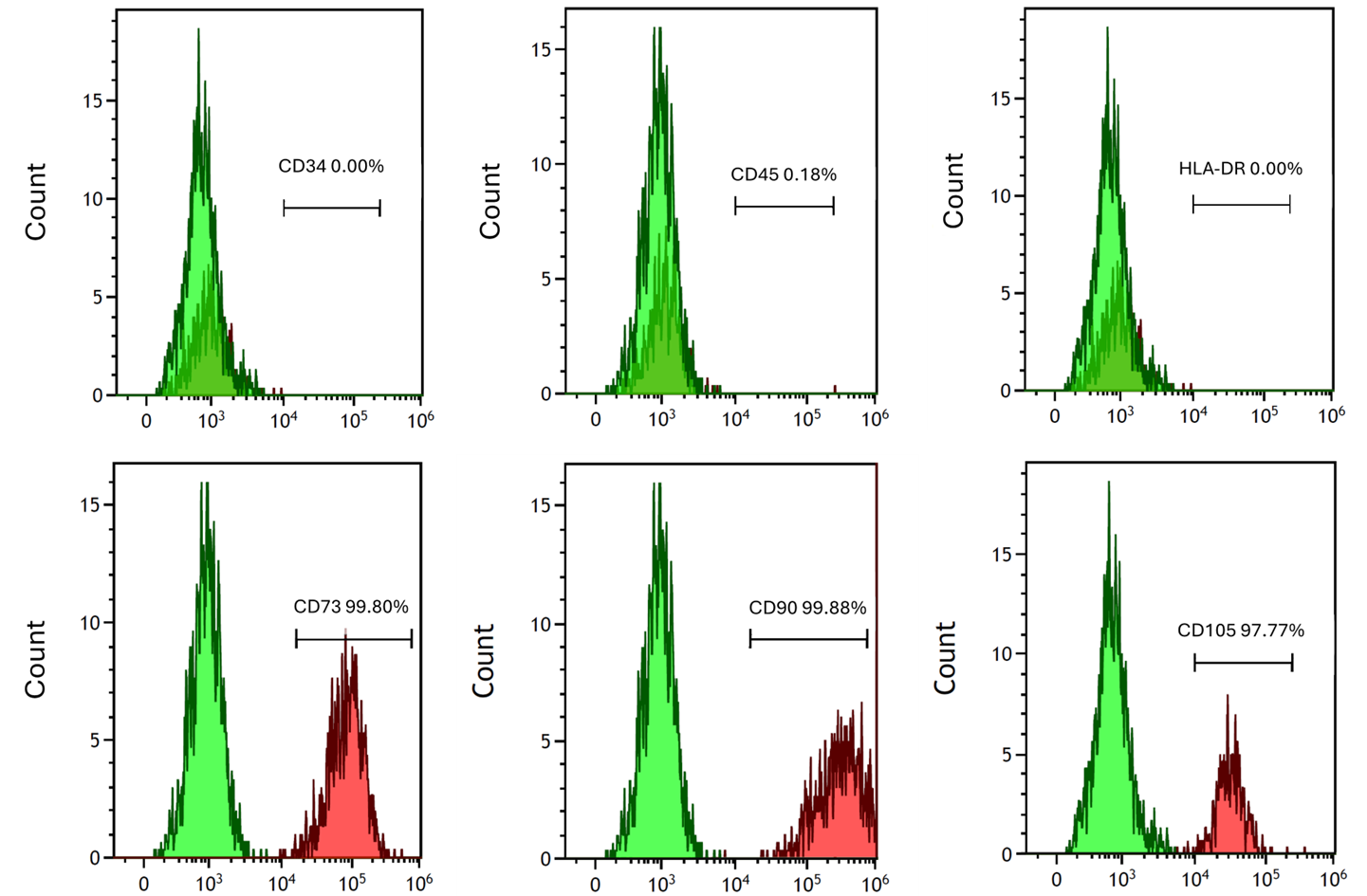
**

**Supplemental Figure 2** The results of the immunophenotyping analysis of ASCs showed expression of specific mesenchymal stem cell surface markers at passage 3 of more than 90%.

**
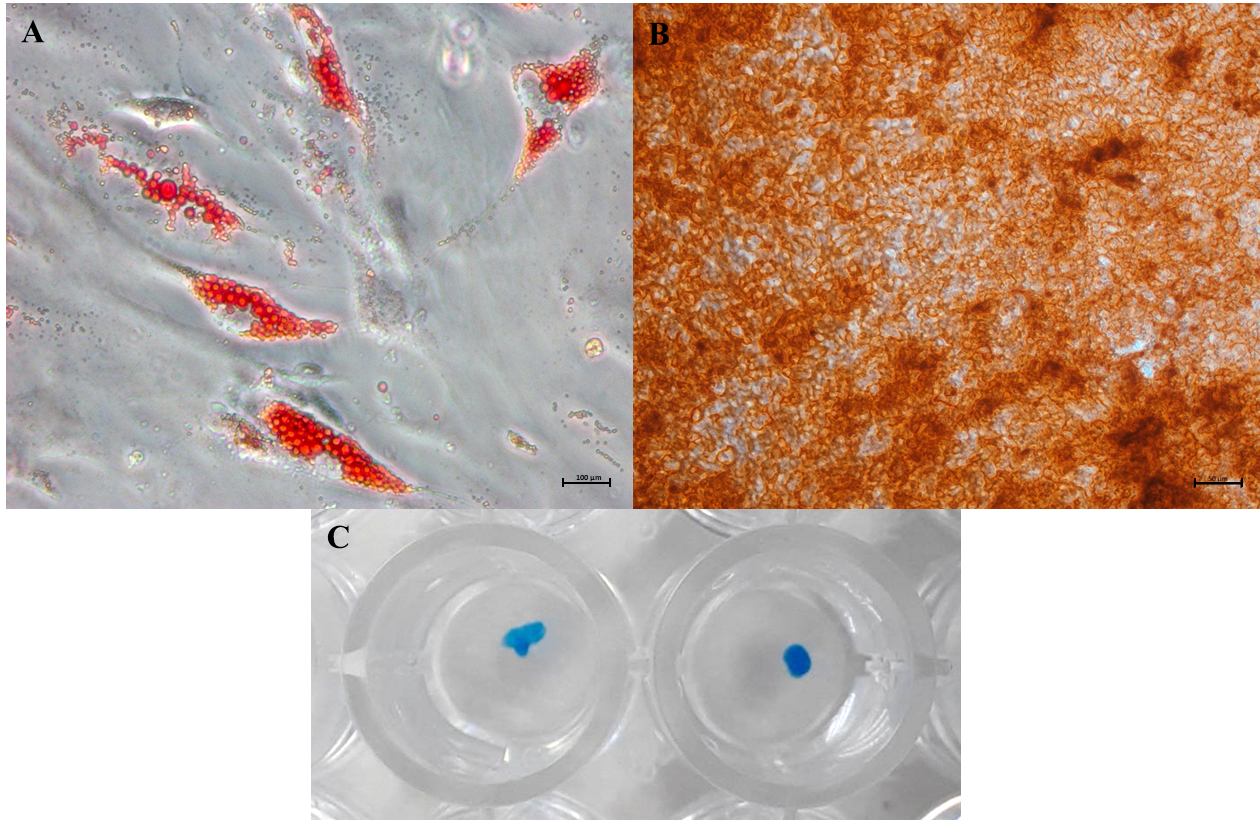
**

**Supplemental Figure 3.** Representative images of ASCs cells differentiated into (A) adipocytes, osteoblasts, and chondrocytes at day 21 in culture, following staining with (A) Oil Red-O; Scale bar = 100 µm, (B) Alizarin Red and; Scale bar = 50 µm, or (C) Alcian blue.
